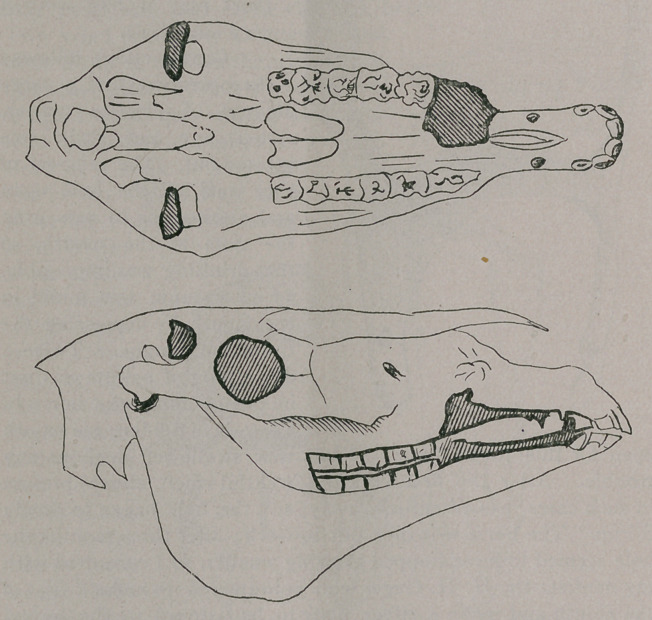# Fistulous Opening from the Mouth

**Published:** 1891-11

**Authors:** Daniel D. Lee

**Affiliations:** Instructor in Anatomy, Veterinary Department, Harvard University


### A FISTULOUS OPENING FROM THE MOUTH, RESULTING FROM A DISEASED TOOTH.

By Daniel D. Lee, M. D. V.,
Instructor in Anatomy,
Veterinary Department, Harvard University.

A bay team horse was brought to me for treatment for roaring. I found the right nasal chamber completely stopped up, the breath very fœtid, a slight bony enlargement on the right side of the nasal peak, the first upper molar almost eaten away by decay and the corresponding lower tooth grown up into the nose.

The history was that the horse had suffered from trouble with his teeth for the last four years and had often changed hands. Two years previously an attempt had been made to remove the upper tooth and some pieces of it were taken out. When trotted the roaring was very loud and signs of asphyxia appeared. Used at a walk or very slow trot he was a good team horse. Next day I cast the horse and removed the lower opposing molar which projected one inch beyond the other lower teeth. I then took out two pieces of the upper molar that had been left from the former operation. One of these pieces was attached to the jaw and the other rode on the crown of the lower molar which projected up from the others; in this way a large opening had been made in the floor of the nasal fossa. I could get my fingers into this opening from the nostril and from the mouth, and was able to remove about two quarts of grain and hay mixed with blood and pus, all in an advanced state of decomposition.

The opening was irregular, and extended from the second upper molar two inches forward, and laterally a little beyond the median line. Not only was the hard palate worn away, but also the lower end of the turbinated bones, and a small hole could be felt through the nasal septum. The great length of the lower tooth and the food forced into the opening, prevented the unattached piece of the upper molar from falling into the mouth; and having great play it had worn a very large hole.

Great relief followed the operation, and a large amount of cheesey matter, rotten hay and grain was snorted out. The removal of this stuff left the hole open and allowed air to pass from the nose to the mouth, so that drinking was impossible, as no vacuum was found in the mouth by depressing the tip of the tongue. Twice a day a plug of oakum was put in the opening, the horse in a few days holding his mouth open for this to be done. Food was swallowed without any trouble. After the first week the frightful smell, always present in such cases, passed entirely away, and the hole began to slowly fill up. The horse was then put to work; after three months the hole seemed to have stopped growing smaller, so I consulted with my dentist, Dr. H. H. Gage, who volunteered to make a cast of the mouth and make a silver plate to be fastened to the second molar by a pivot. We cast the horse and after one or two trials Dr. Gage was able to get an exact plastic cast of the hole and surrounding tissues. From this he made a metal cast on which he beat out the silver plate, a drawing of which is given above.

After the plate was finished we cast the horse and found that as far as it went the plate was perfect, but that the drilling of the second upper molar for the rivet was very hard. However, after making a small incision in the cheek in which we put a short tube to keep the tissues off the twist drill, we went to work. Seven steel twist-drills were broken, two of them remaining in the tooth, so we had to abandon our attempts. During the operation the horse felt no pain, as all the prejecting part of the teeth is solid, the pulp cavity being ossified. A few days after this the horse had a severe nose bleed while plowing, and thinking that he had done enough in this world, I sent him to the next.

Below are two views of his skull, the opening being shown in each in black; they give a better idea of the condition than a written description.

I know of one similar case where death resulted from inability to swallow after removal of a tooth that projected up into the nose, but bony disease was also present and almost the whole palate eaten away. I have also heard of two cases of openings from mouth to nose after removal of diseased upper molars, but they were small and soon filled up. The same condition is sometimes seen in young animals, in whom the closure of the suture of the palate is not complete ; in these cases the greater part of the milk taken from the mother is lost from imperfect deglutition. If I ever meet with a similar case I hope to be able to insert a plate successfully.

I think this can be done *provided* no disease involving the bone cause the trouble with the teeth. I have two skulls, in the museum of the Veterinary School, where the teeth have been lost from cancerous disease which involved the bones of the upper jaw. Such cases are of course hopeless.

## Figures and Tables

**Figure f1:**
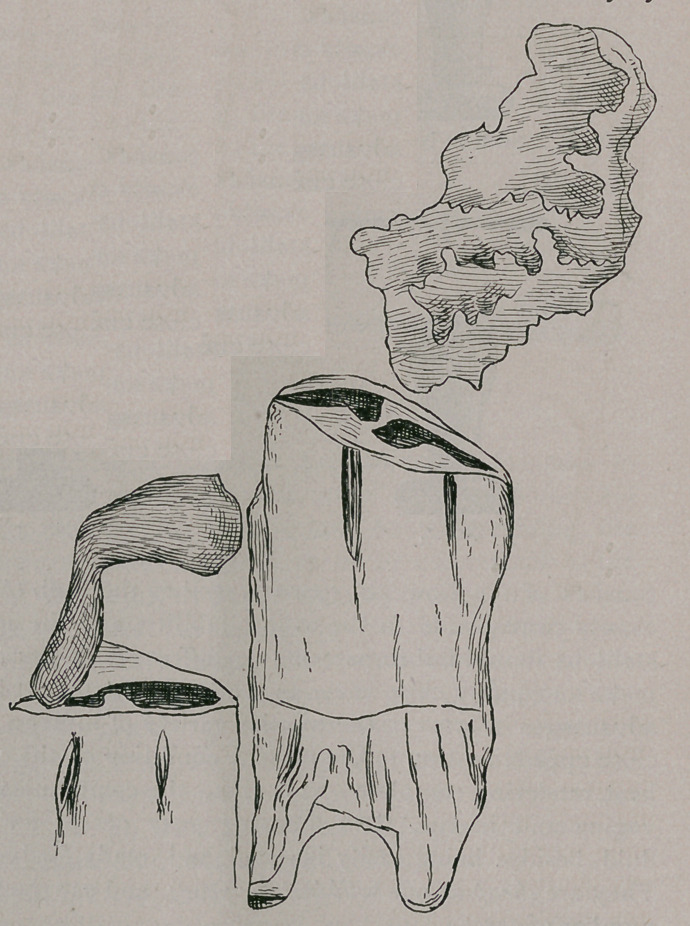


**Figure f2:**
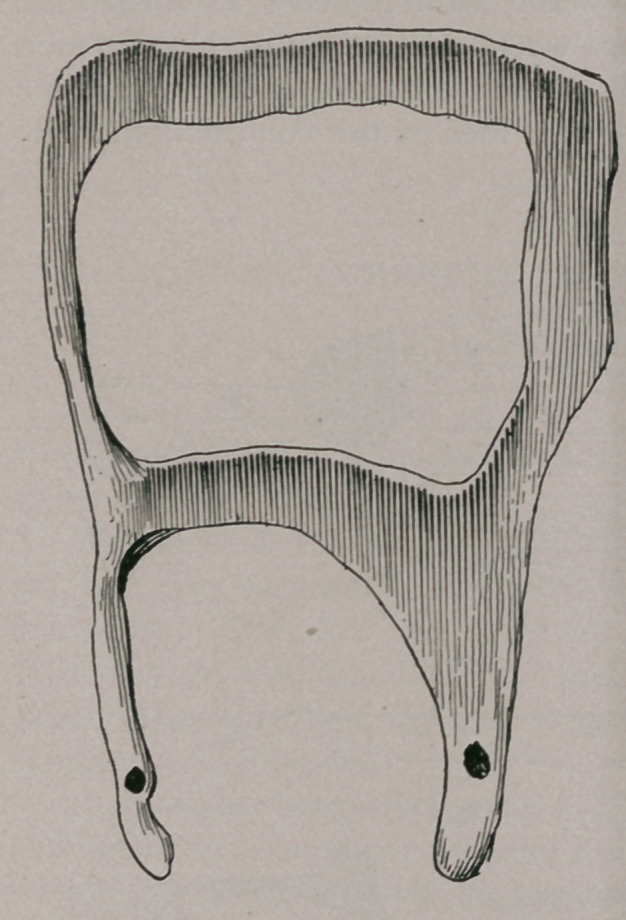


**Figure f3:**